# Novel electrical and mechanical characteristics of composites composed of electrically conducting Ni-Cr alloy particles in non-conducting soda-lime glass

**DOI:** 10.1038/s41598-017-15213-y

**Published:** 2017-11-07

**Authors:** Yoshiharu Waku, Teruyuki Yamashita, Hiroyuki Kitagawa, Masahiro Yoshinobu, Hiromichi Katsuyama, Daiki Hamano, Shinji Harui

**Affiliations:** 1 Interdisciplinary Graduate School of Science and Engineering, Shimane University, Matsue, Shimane 690-8504 Japan; 2 Suzuki Gokin Co., Ltd., 2-5-27 Tsurumachi, Taisho-ku, Osaka, 551-0023 Japan; 3 Present Address: Department of Material Science, Graduate School of Engineering, Tohoku University, Sendai, Miyagi 980-8573 Japan; 4 Present Address: UBE Machinery Corporation Ltd., Ube, Yamaguchi, 755-8633 Japan

## Abstract

The bulk resistivity of Ni-Cr alloys is inherently constant. Therefore, it is necessary to regulate the cross-section and length of Ni-Cr alloys to achieve the desired electrical resistance. Here, we describe a composite, comprising a soda-lime glass matrix and disk-like Ni-Cr alloy particles, that has variable bulk resistivity. The bulk resistivity of the composite can be controlled accurately by adjusting the volume fraction (30–80 vol% Ni-Cr alloy) and aspect ratio of the particles. Furthermore, the composite’s strength and fracture toughness are both improved by microdispersion of the disk-like Ni-Cr alloy particles. A 1/100-scale model resistor based on this composite was found to have attractive properties for electrical power applications, such as an approximately 50% reduction in volume, a 65% reduction in weight, and a 95% reduction in inductance in comparison with a conventional neutral grounding resistor made from special cast iron. Clearly, use of this composite material for fabrication of ubiquitous electrical components would greatly reduce the demand and consumption of Ni and Cr for this application. Several benefits are envisioned from this development, including the fabrication of downsized devices and the availability of lower-cost home appliances and industrial products.

## Introduction

Ni-Cr alloys are widely used as electrical resistance wires in many home appliances and various kinds of industrial equipment in high-temperature oxidising environments because of their Cr-induced anti-corrosion properties^1–3^. To apply Ni-Cr alloys to a resistor or heating element, however, it is necessary to adjust the electrical resistance by secondary processing. The ohmic resistance, *R*, of a resistor is given by *R* = *ρ* (*L*/*A*), where *ρ* is the bulk resistivity, *L* is the length, and *A* is the cross-sectional area of the material. Therefore, *R* is controlled by *L* and *A*, given that *ρ* for conventional metallic resistance is constant. Consequently, various types of coils made from Ni-Cr alloy wires are fabricated by secondary processing to attain the desired ohmic resistance. If composites that can control bulk resistivity can be developed, they will be expected to find a wide range of uses as resistors or heating elements.

The electrical conductivity of insulating materials can be increased by the addition of conductive materials in particulate form^4^. Therefore, electrical percolation in mixtures of electrically conducting and non-conducting materials has been widely investigated for conductive polymers. The electrical percolation threshold for the majority of polymer/carbon nanotube composites has been found to be less than 5 wt%^5,6^. Lower filler levels allow for an increase in electrical conductivity without significantly reducing the flexibility of the polymer^6^. However, these materials are not considered suitable for applications as resistors or heating elements.

To improve the fracture toughness of brittle materials, dispersing ceramic particles or whiskers, etc., with the intention of using mechanisms such as phase transformation, micro cracking, crack deflection, pull-out, bridging, and shielding effects of residual compressive stress has, to date, been applied to brittle materials such as ceramics and glasses^7^. However, these improvements have not been prominent; that is, no effective technique has been found for significant toughness improvement.

One approach to improve fracture toughness has been reported that involves the uniform microdispersion of flaky ductile metallic particles in Al_2_O_3_ ceramics^8^ and MAS glass^7,9^. The fundamental concept of this approach is based on increasing the plastic energy and surface energy in the Griffith-Irwin theoretical equation at the time of fracture. This technique has the effect of improving fracture toughness by increasing plastic energy, γ_p_, through the plastic deformation of metallic particles at a crack tip^7^.

In this work, to develop novel composites that can control bulk resistivity, soda-lime glass matrix composites reinforced with disk-like Ni-Cr alloy particles were fabricated by spark plasma sintering (SPS) and their electrical and mechanical properties were evaluated. In addition, the possibility of their application as an electrical power resistor is discussed.

## Results

### Configuration of particles and microstructures of composites

**Figure 1:**
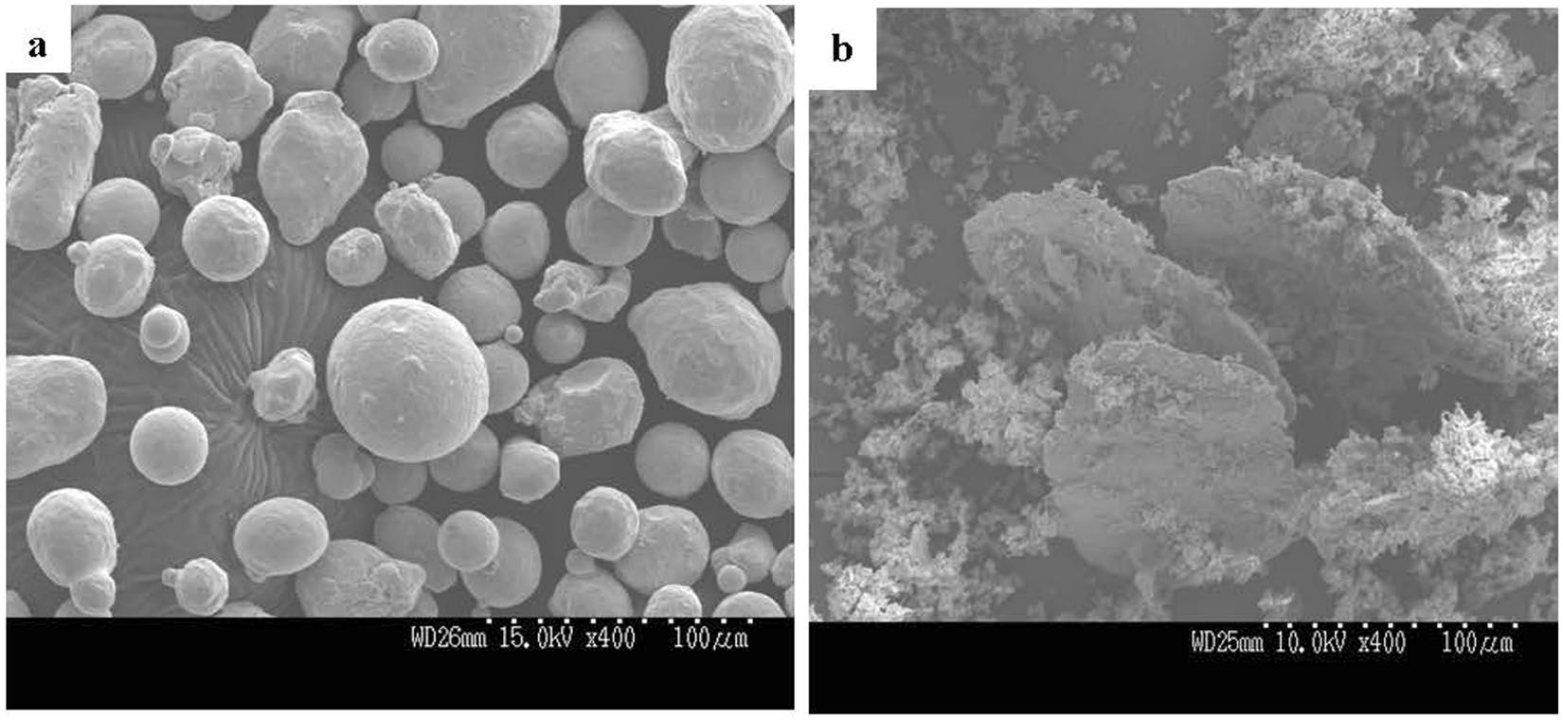
SEM images of as-received powder and disk-like particles. (**a**) SEM images showing as-received Ni-Cr alloy powder. (**b**) Disk-like Ni-Cr alloy particles fabricated by wet planetary ball milling at  309 rpm for 10 h.

Figure 1a displays a scanning electron microscopy (SEM) image of the as-received Ni-Cr alloy powder. Figure 1b shows disk-like Ni-Cr alloy particles obtained by wet planetary ball milling at 309 rpm for 10 h. From Fig. 1a and b, before ball milling, the Ni-Cr alloy particles were spherical; afterwards, as shown in Fig. 1b, the Ni-Cr particles were plastically deformed to a disk-like shape and pulverised soda-lime glass powder adhered to its surface.

**Figure 2:**
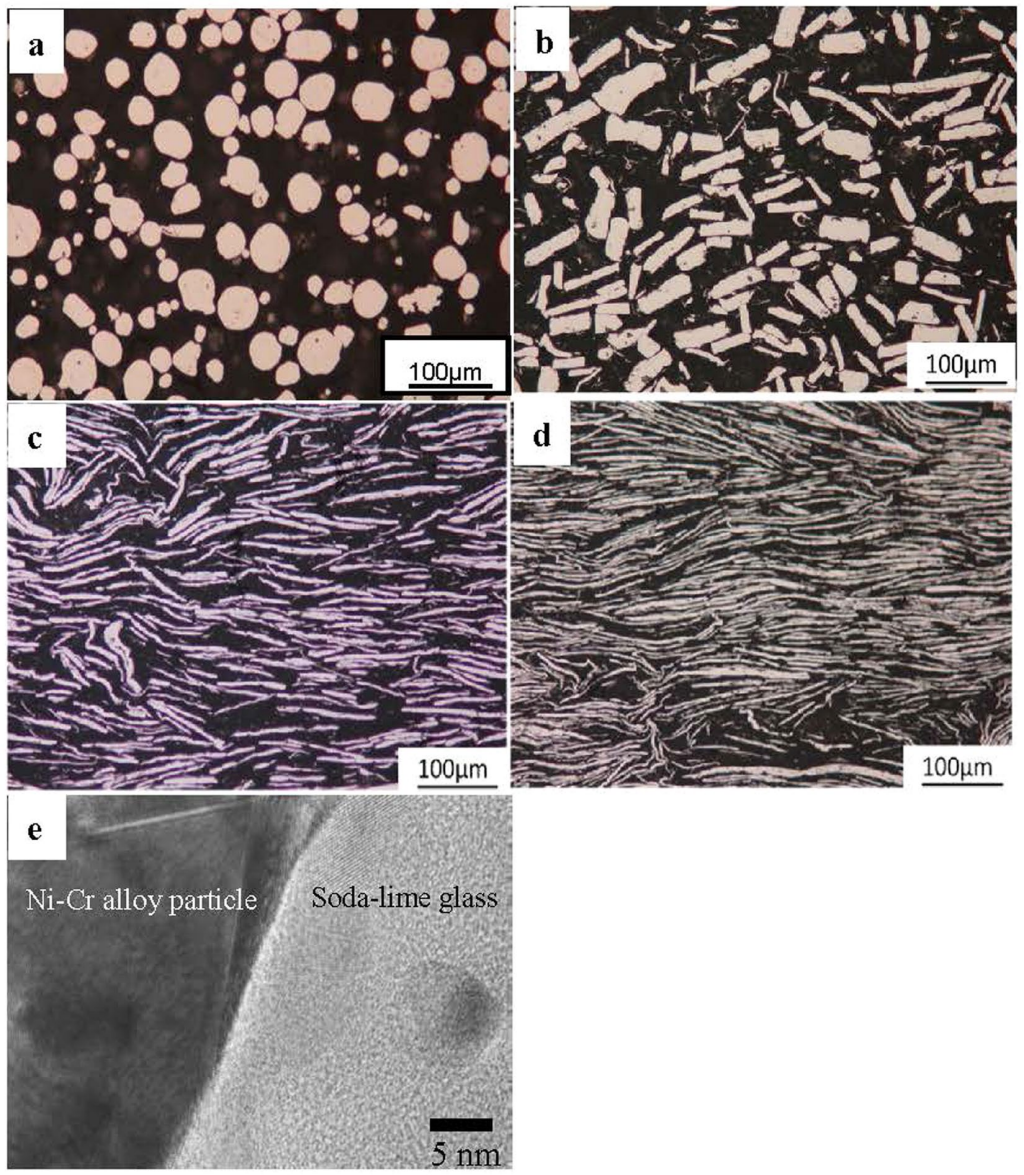
Optical micrographs of microstructures of composites and HRTEM image of interface. (**a**–**d**) Optical micrographs showing the microstructure of a cross-section perpendicular to the SPS-pressed planes of the soda-lime glass/30 vol% Ni-Cr alloy particle composites. Spherical (**a**) and disk-like (**b–d**) Ni-Cr alloy particles in the composites obtained by wet planetary ball milling at (**a**) 182 rpm for 30 min, (**b**) 273 rpm for 10 h, (**c**) 309 rpm for 10 h, and (**d**) 345 rpm for 10 h. (**e**) HRTEM image of the interface between the soda-lime glass and a disk-like Ni-Cr alloy particle in the specimen shown in Fig. 2c. The beam direction is that of <110> Ni.

Figure 2 shows optical micrographs of the microstructures perpendicular to the spark plasma sintering (SPS) pressed plane of the soda-lime glass/30 vol% Ni-Cr alloy particle composites. In the figures, the light areas in the microstructures represent the Ni-Cr alloy particles and the black areas are the soda-lime glass matrix. After the Ni-Cr alloy particles were milled at 182 rpm for 30 min, the particles were virtually the same as those in the “as-received” condition (Fig. 2a). From Fig. 2b–d, massive or disk-like Ni-Cr alloy particles were comparative uniformly dispersed within the soda-lime glass matrix. For the disk-like Ni-Cr alloy particles (Fig. 2c and d), longitudinal sections of disk-like Ni-Cr alloy particles showed a tendency to align perpendicular to the SPS pressing direction and were widely observed in cross-sections of the microstructure perpendicular to SPS pressed planes.

Disk-like forming behaviour becomes increasingly apparent with increases in both the revolution speed of the wet planetary ball mill and milling time. The aspect ratio of disk-like Ni-Cr alloy particles can be evaluated by measuring the ratio of the length of the major axis to the length of the minor axis in the circumscribed rectangles in each particle. It is clear that the aspect ratio of Ni-Cr alloy particles increased with increasing revolution speed of the planetary ball mill (at 273 rpm for Fig. 2b, at 309 rpm for Fig. 2c, and at 345 rpm for Fig. 2d). Conversely, the spacing between disk-like Ni-Cr alloy particles decreased with increasing revolution speed of the planetary ball mill. The spacing between disk-like Ni-Cr alloy particles tends to be smaller than that between spherical Ni-Cr alloy particles.

As can be seen from HRTEM images of the interface between disk-like Ni-Cr alloy particles and the soda-lime glass matrix shown in Fig. 2e, no reaction phases were observed at the interface, which is relatively well connected. Secondary cracks are frequently observed at the interface between disk-like Ni-Cr alloy particles and the matrix, which indicates that the bonding strength of the interface is not very high; thus, the interface may act as an effective path for crack propagation.

### Influence of aspect ratio and relative volume on bulk resistivity

**Figure 3:**
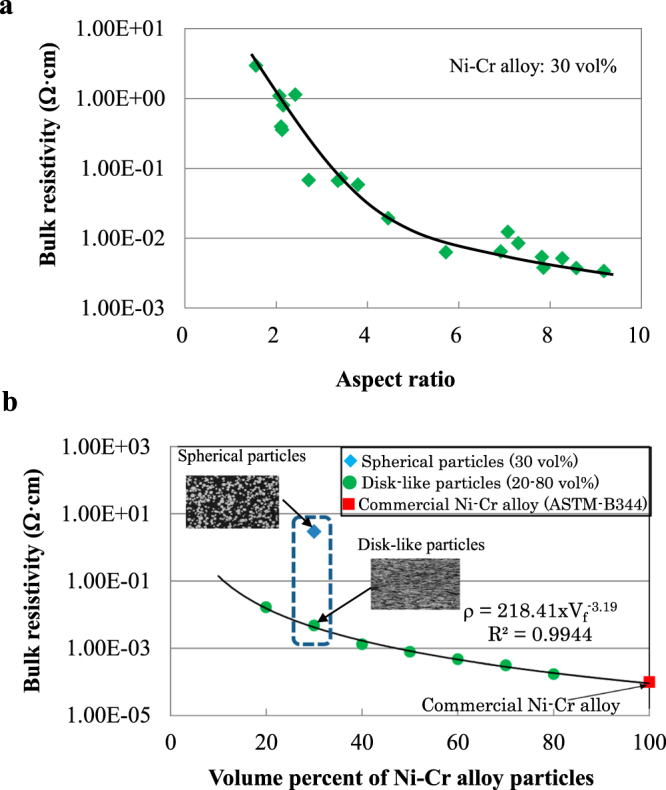
Change in bulk resistivity with respect to relative volume and aspect ratio. (**a**) Dependence of the bulk resistivity on the aspect ratio of the disk-like Ni-Cr alloy particles for soda-lime glass/30 vol% Ni-Cr alloy particle composites. (**b**) Relationship between bulk resistivity and relative volume of the disk-like Ni-Cr alloy particles for soda-lime glass/Ni-Cr alloy particle composites, compared with a composite containing 30 vol% spherical Ni-Cr alloy particles.

Figure 3a shows the effect of the aspect ratio of the Ni-Cr alloy particles on the bulk resistivity at room temperature for soda-lime glass/30 vol% Ni-Cr alloy particle composites. The aspect ratio of the Ni-Cr alloy particles was controlled by careful selection of experimental conditions for wet planetary ball milling (i.e., 182 rpm for 30 min or 182–345 rpm for 10 h). The bulk resistivity of the composites decreased rapidly as the aspect ratio of the Ni-Cr alloy particles increased to approximately 5, and then decreased slowly until the aspect ratio reached 9.2. At an aspect ratio of 2.0, the bulk resistivity at room temperature was 1.0 Ω·cm, whereas at an aspect ratio of 8.57, the bulk resistivity was approximately 250 times smaller, 4.0 × 10^−3^ Ω·cm.

Figure 3b shows the relationship between the bulk resistivity and the relative volume of the disk-like Ni-Cr alloy particles in the soda-lime glass/disk-like Ni-Cr alloy particle composites compared with a composite containing 30 vol% spherical Ni-Cr alloy particles. The disk-like Ni-Cr alloy particles were obtained by ball milling at 309 rpm for 10 h. The solid line in the figure can be expressed as *ρ* = 218.41 × V_f_^−3.19^, where *ρ* is the bulk resistivity and V_f_ is the relative volume of the disk-like Ni-Cr alloy particles. For composites containing 30 vol% Ni-Cr alloy particles (the blue dotted line in Fig. 3b), there was a marked difference in bulk resistivity between spherical particles and the disk-like particles. The bulk resistivity of 5.14 × 10^−3^ Ω·cm in the composite reinforced with disk-like Ni-Cr alloy particles was approximately 0.17% that for the composite reinforced with spherical Ni-Cr alloy particles, i.e., 2.95 Ω·cm. At the same relative volume of Ni-Cr alloy particles, therefore, the bulk resistivity of soda-lime glass/Ni-Cr alloy particle composites was greatly influenced by the configuration of Ni-Cr alloy particles.

The bulk resistivity of the soda-lime glass/disk-like N-Cr alloy particle composites decreased with increasing relative volume of disk-like Ni-Cr alloy particles and gradually approached the bulk resistivity value of a commercial Ni-Cr alloy (ASTM-B344). Therefore, the bulk resistivity of the composite containing disk-like Ni-Cr alloy particles may be controlled precisely by an appropriate combination of relative volume and aspect ratio of disk-like Ni-Cr alloy particles. Conventional coils or even complex-shaped resistive materials could be replaced with a sheet of this composite material. In the case of resistance heating, for example, the composite could serve as a sheet heater with uniform heat distribution. In addition, in case of applying the composite reinforced with disk-like Ni-Cr alloy particles to resistors or heating elements, the required amount of Ni and Cr will be greatly reduced in comparison with that of a commercial Ni-Cr alloy.

It has been reported that nanometre-size particles exhibit peculiar behaviour quite different from that of a bulk material because of surface or quantum confinement effects^10,11^. The particles used in this study have a specific “disk-like” shape, but the particle size is in the classical regime, not the nanometre scale.

### Temperature dependence of resistance (TCR)

**Figure 4:**
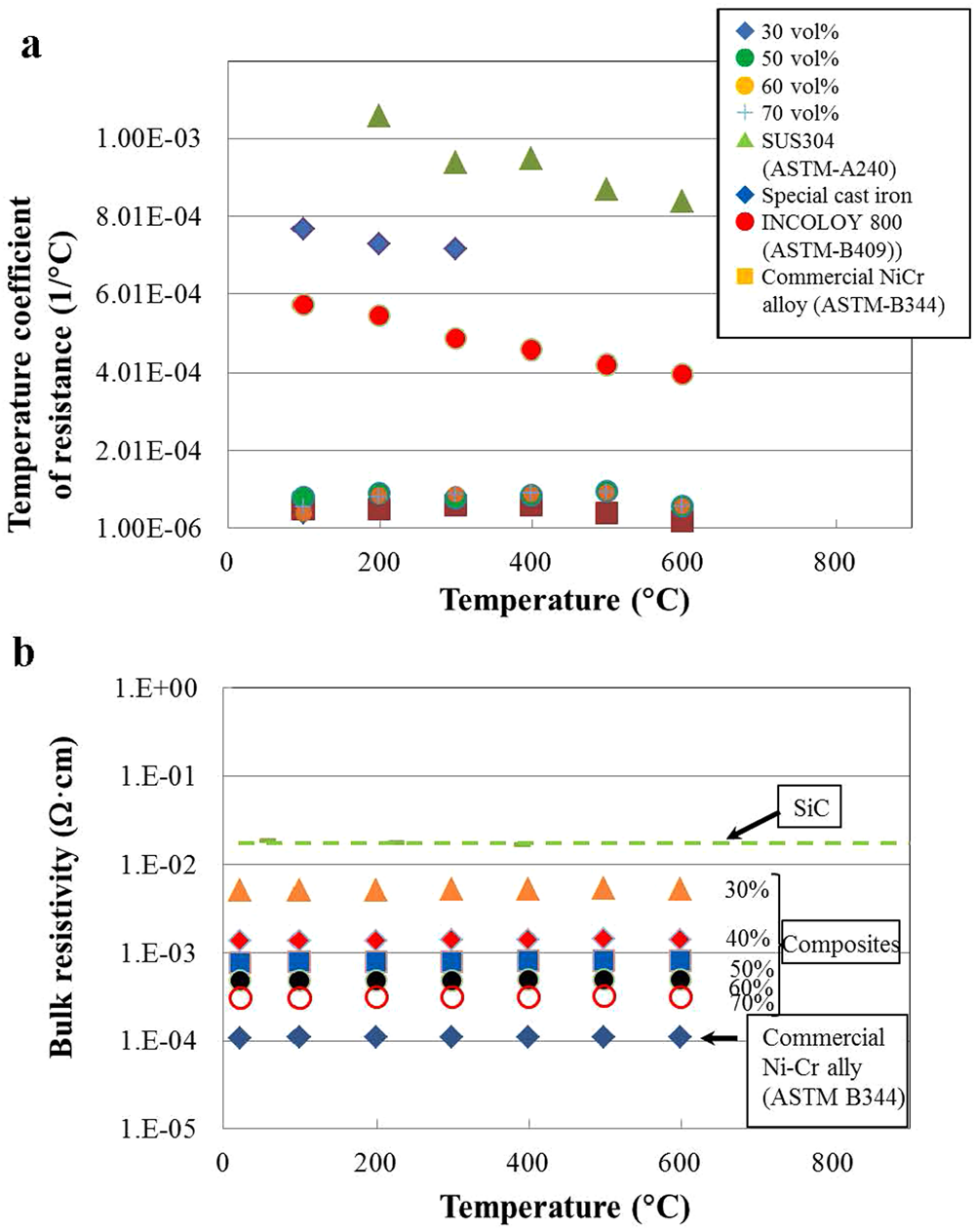
Temperature dependence of bulk resistivity of composites. (**a**) Dependence of TCR on temperature for soda-lime glass/30–70 vol% disk-like Ni-Cr alloy particle composites, compared with that for SUS304, a special cast iron, INCOLOY 800, and a commercial Ni-Cr alloy. (**b**) Change in the bulk resistivity with increasing temperature for soda-lime glass/30–70% disk-like Ni-Cr alloy particle composites, compared with commercial Ni-Cr alloys and SiC ceramics.

Figure 4a shows the dependence of the temperature coefficient of resistance (TCR) on temperature for the soda-lime glass/disk-like Ni-Cr alloy particle composites, compared with a commercial Ni-Cr alloy (ASTM-B344), the stainless steel SUS304 (ASTM-A240), a special cast iron (Catalogue, Suzuki Gokin Co., Ltd., Osaka, Japan, use in the production of the 1/100-scale neutral grounding resistor for evaluation of resistor performance), and INCOLOY 800 (ASTM-B409). The TCRs of SUS304, the special cast iron, and INCOLY 800 showed a slight temperature dependence, decreasing with an increase in temperature. Meanwhile, the TCRs of composites (in the range of 30–70 vol% disk-like Ni-Cr alloy particles) showed little temperature dependence, with nearly the same values as a commercial Ni-Cr alloy. Thus, the TCRs of composites can be considered to be controlled by the TCRs of the reinforcing particles.

Figure 4b shows changes in bulk resistivity as a function of temperature for composites containing 30%, 40%, 50%, 60%, and 70% disk-like Ni-Cr alloy particles, in comparison with those for a SiC ceramic heater material and a commercial Ni-Cr alloy. The bulk resistivity of composites at a given relative volume of alloy particles, a commercial Ni-Cr alloy, and SiC ceramic heater was approximately constant from room temperature to 600 °C. The bulk resistivity of composites, however, changed with the relative volume of disk-like Ni-Cr alloy particles: as the relative volume of alloy particles in the composite increased, the bulk resistivity of the composite decreased. The bulk resistivity of the composites is distributed between that of a commercial Ni-Cr alloy and that of SiC ceramics.

We can therefore control the bulk resistivity of composites, ranging from that of a commercial Ni-Cr alloy to that of SiC ceramics, by adjusting the relative volume of disk-like Ni-Cr alloy particles.

### Flexural strength and fracture toughness

**Figure 5:**
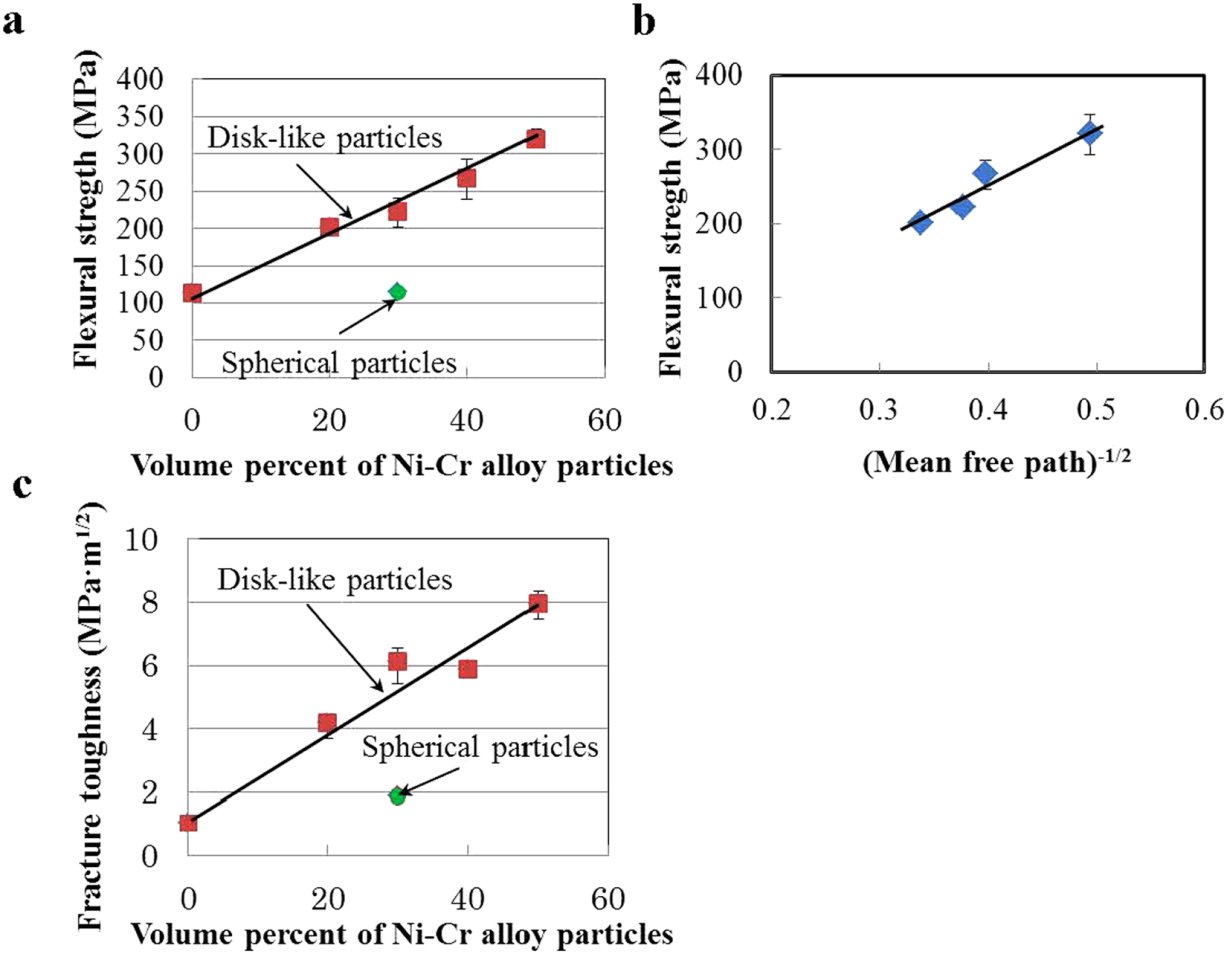
Flexural strength and fracture toughness of composites. (**a**) Relationship between flexural strength and relative volume of disk-like Ni-Cr alloy particles. (**b**) Relationship between flexural strength and (particle mean free path)^−1/2^ of disk-like Ni-Cr alloy particles in soda-lime glass/disk-like Ni-Cr alloy particle composites. (**c**) Dependence of fracture toughness on relative volume of disk-like Ni-Cr alloy particles for soda-lime glass/disk-like Ni-Cr alloy particle composites.

Figure 5a shows the relationship between flexural strength and relative volume of disk-like Ni-Cr alloy particles for soda-lime glass/disk-like Ni-Cr alloy particle composites. For comparison, the flexural strength of soda-lime glass/30 vol% spherical Ni-Cr alloy particle composites is shown in Fig. 5a. The disk-like Ni-Cr alloy particles were obtained by wet planetary ball milling at 309 rpm for 10 h. For the composite with disk-like Ni-Cr alloy particles, the flexural strength increased markedly with increasing relative volume of disk-like Ni-Cr alloy particles; at 50 vol% disk-like Ni-Cr alloy particles, the flexural strength increased to 321 MPa, approximately 2.8 times the value (113 MPa) for monolithic soda-lime glass. In contrast, for the composite with 30 vol% spherical Ni-Cr alloy particle, the flexural strength was 114 MPa, which is nearly the same value as that for the monolithic soda-lime glass. Thus, the effect of Ni-Cr alloy particles on the flexural strength differed greatly, depending on the shape of Ni-Cr alloy particles at a given volume fraction of Ni-Cr alloy particles.

The spacing between the disk-like Ni-Cr alloy particles decreased with increasing relative volume of disk-like Ni-Cr alloy particles. Figure 5b shows the relationship between σ_b_ and λ^−1/2^, which is fairly linear (σ_b_ and λ are the flexural strength of composites and the mean free path of disk-like Ni-Cr alloy particles, respectively). Figure 5b demonstrates that the flexural strength can be expressed as a function of λ^−1/2^, which is similar to the Hall-Petch equation.

**Figure 6:**
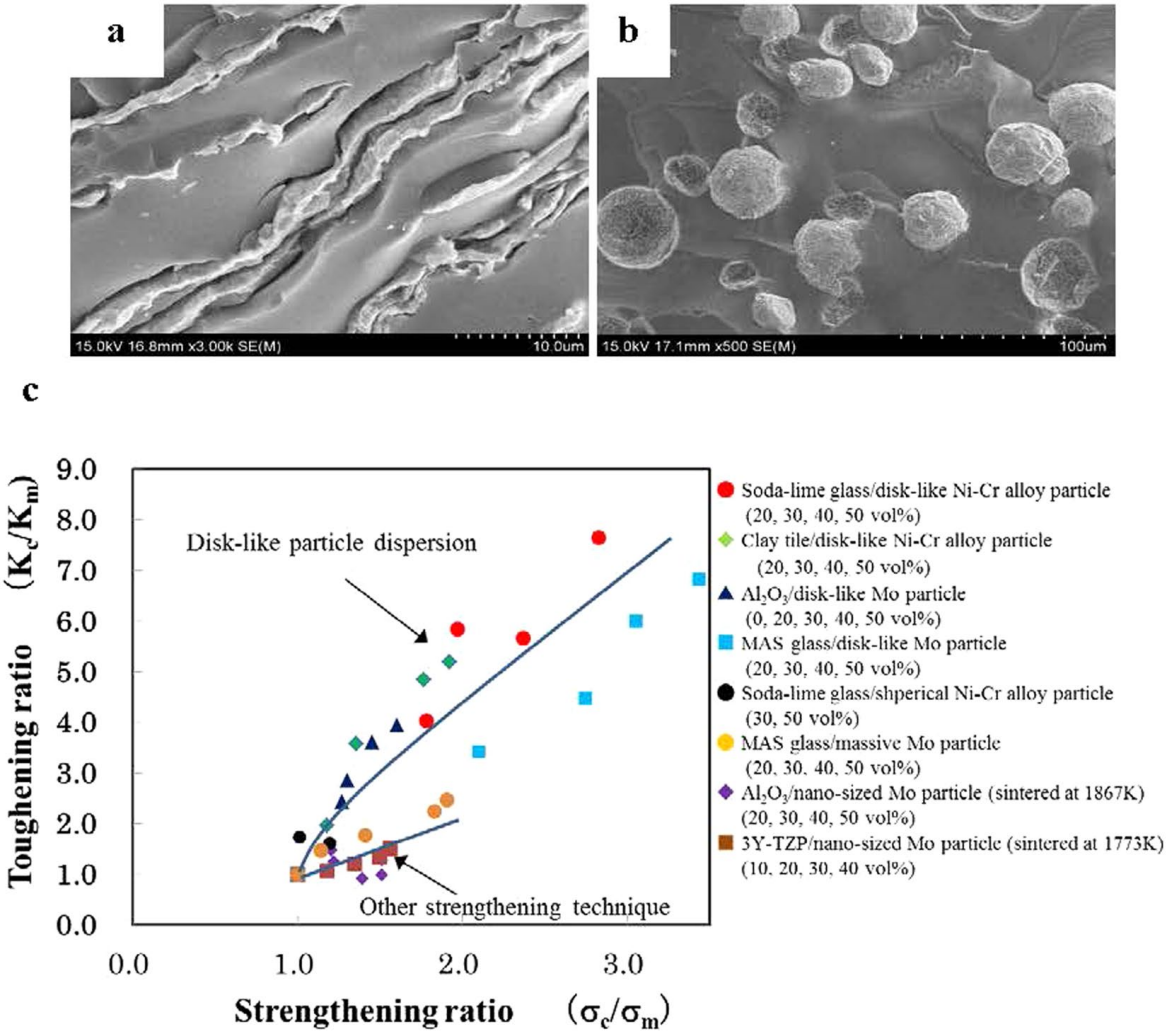
SEM images of fracture surface and relationship between strengthening ratio and toughening ratio. (**a**) and (**b**) SEM images of the fracture surface of soda-lime glass/30 vol% Ni-Cr alloy particle composites. (**a**) Disk-like Ni-Cr alloy particles. (**b**) Spherical Ni-Cr alloy particles. (**c**) Comparison of the relationship between strengthening ratio and toughening ratio for composites made of soda-lime glass/spherical Ni-Cr alloy particles and soda-lime glass/disk-like Ni-Cr alloy particles, and composites using other strengthening techniques.

Figure 5c shows the dependence of fracture toughness on the relative volume of disk-like Ni-Cr alloy particles. For comparison, the fracture toughness of a soda-lime glass/30 vol% spherical Ni-Cr alloy particle composite is shown in Fig. 5c. The fracture toughness of the composites containing disk-like Ni-Cr alloy particles increased with increasing relative volume of disk-like Ni-Cr alloy particles. The fracture toughness of composites with 30 vol% disk-like Ni-Cr alloy particles was 6.01 MPa·m^1/2^, approximately 3.2 times higher than the value (1.87 MPa·m^1/2^) for composites with 30 vol% spherical Ni-Cr alloy particles. At 50 vol% of disk-like Ni-Cr alloy particles, the fracture toughness greatly increased to 7.94 MPa·m^1/2^, approximately 7.6 times higher than that of monolithic soda-lime glass (1.04 MPa·m^1/2^). For spherical Ni-Cr alloy particles, the fracture toughness of the composite containing 30 vol% Ni-Cr alloy particles was 1.87 MPa·m^1/2^, slightly higher than the value (1.04 MPa·m^1/2^) for monolithic soda-lime glass. The slight improvement of the fracture toughness can be interpreted as crack deflection caused by spherical particles, as seen in Fig. 6b. The ratio of fracture toughness improvement differs greatly with respect to the particle shape of the Ni-Cr alloy, even when the same Ni-Cr alloy particles are used as starting materials, with disk-like particles being much more effective.

Fracture toughness, *K*_1C_, can be related to plastic energy, *γ*_p_, as *K*_1C_ = (Eγ_p_)^1/2^. Figure 6a and b show SEM images of a fracture surface of composites made of soda-lime glass/disk-like Ni-Cr alloy particles (Fig. 6a) and soda-lime glass/spherical Ni-Cr alloy particles (Fig. 6b) with 30 vol% Ni-Cr alloy tested for fracture toughness. In the case of spherical Ni-Cr alloy particles, in which improvement of the fracture toughness is relatively slight, there are not many traces of plastic deformation of Ni-Cr alloy particles in the fracture surface. It is deduced from Fig. 6b that cracks propagate along the interface between spherical Ni-Cr alloy particles and the matrix. The fracture toughness increase results primarily from crack deflection and pull-out of Ni-Cr alloy particles from the matrix. In contrast, for the case of disk-like Ni-Cr alloy particles, there is noticeable plastic deformation of disk-like Ni-Cr alloy particles in the fracture surface, and an increase in plastic energy may increase the fracture toughness. This may be the main cause of the increase in the fracture toughness dependence on the relative volume of Ni-Cr alloy particles seen in Fig. 5c.

With respect to the toughening of brittle materials by crack-tip blunting using a ductile phase, Cahn^12^ has introduced the toughening ratio, λ_b_, which is defined as the ratio of the applied stress intensity factor, K_c_, to that in the matrix, K_m_. Figure 6c displays the relationship between the toughening ratio and strengthening ratio for composites made of soda-lime glass/disk-like Ni-Cr alloy particles and soda-lime glass/spherical Ni-Cr alloy particles. The strengthening ratio is defined as the ratio of the composite flexural strength to the matrix flexural strength. For comparison, the relationship between the toughening ratio and strengthening ratio for a clay tile/disk-like Ni-Cr alloy particle composite, an Al_2_O_3_/disk-like Mo particle composite^8^, a MgO-Al_2_O_3_-SiO_2_ glass/Mo particle composite^7,9^, and microdispersions of massive Mo particles^7^ and nano-sized Mo particles^13,14^ are shown.

Figure 6c shows that dispersions of spherical Ni-Cr alloy particles, massive Mo particles, and nano-sized Mo particles are effective for increasing the strength, but not very effective for increasing the fracture toughness. In cases where the dispersed phase is increased, there is almost no plastic deformation, and the main mechanisms responsible for the increase in toughness are crack deflection effects, crack shielding effects, etc. In addition, strength is increased effectively, but no drastic increase in toughness can be expected. In contrast, microdispersing the disk-like Ni-Cr alloy particles is effective for simultaneously increasing strength and fracture toughness and can be considered a new toughening technique for brittle materials such as glass and ceramics.

### Evaluation of resistor performance

**Figure 7:**
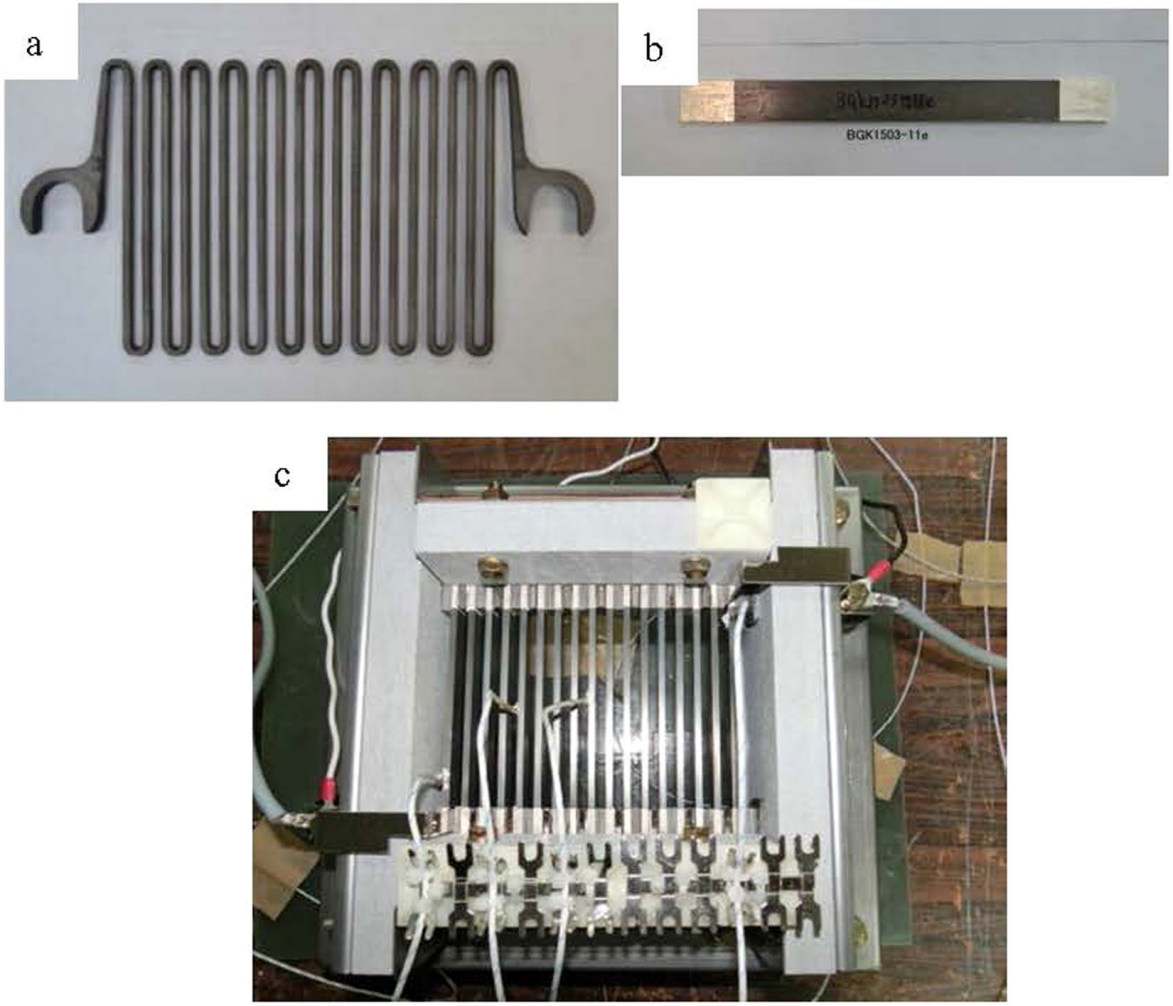
Resistance materials and 1/100-scale model of resistor. (**a**) Conventional resistance materials for the special cast iron resistor. (**b**) The present composite resistance material. (**c**) 1/100-scale model resistor made of the present composites for electrical power applications.

To confirm the suitability of the present composites for use as electrical power resistors, a 1/100-scale model resistor was fabricated using the soda-lime glass/35 vol% disk-like Ni-Cr alloy particle composite, and the performance was compared with that of a 1/100-scale neutral grounding resistor composed of a special cast iron resistor. The conventional resistance material of the special cast iron is strip-shaped, as seen in Fig. 7a. In contrast, the soda-lime/35 vol% disk-like Ni-Cr alloy particle composite resistance is in the form of a simple sheet, as shown in Fig. 7b

Figure 7c shows the 1/100-scale model resistor made of the present composite (Fig. 7b) as an electrical power resistor; the rating specifications of the resistor were 66/√3 kV, 100 A, and 381 Ω. In comparison with the conventional neutral grounding resistor, there was a significant improvement in key values, such as an approximately 50% reduction in volume, a 65% reduction in weight, and a 95% reduction in inductance.

The three main characteristics of the soda-lime glass/disk-like Ni-Cr alloy particle composites are as follows. The bulk resistivity can be controlled precisely by altering the combination of the relative volume and the aspect ratio of the particles. The flexural strength and fracture toughness of brittle materials can be increased simultaneously by microdispersion of disk-like Ni-Cr alloy particles. The use of composites in electrical resistance or heater materials will greatly reduce the required amount of expensive Ni and Cr. As a consequence, such composites may find use in diverse applications, including household electrical appliances and new sheet heaters that can heat up uniformly, instead of coil heaters.

## Methods

### Material preparation

Commercially available soda-lime glass powder with an average particle size of 14 μm (B200, Nissho-materials Co., Ltd., Mie, Japan) was used as the matrix, and an 80% Ni-20% Cr alloy with a size range of 20–53 μm (1616-02, Höganäs Belgium SA, Ath, Belgium) was used as the metallic alloy powder.

The effect of the relative volume of Ni-Cr alloy particles on the electrical and mechanical properties of soda-lime glass/Ni-Cr alloy particle composites was studied in composites containing 20, 30, 40, 50, 60, 70, and 80 vol% particles. Ni-Cr alloy particles were milled using a wet planetary ball mill (Pulverisette-6, Fritsch GmbH, Idar-Oberstein, Germany) with Si_3_N_4_ balls in ethanol for 10 h to obtain disk-like particles and a homogeneous mixture of soda-lime glass powder and Ni-Cr alloy powder. The slurry was dried in a rotary evaporator to remove the ethanol. The mixture was sintered by SPS (Plasman CSP-KIT-0212, S.S. Alloy Co., Ltd., Hiroshima, Japan) at 973 K under 38 MPa and pressed in a carbon die to fabricate ϕ10 × 1.5 mm composites to evaluate the bulk resistivity. To measure the three-point flexural strength and fracture toughness, composites with dimensions of 70 × 70 × 5 mm were fabricated by SPS (SPS3.20MK-IV, Sumitomo Coal Mining Co., Ltd., Tokyo, Japan) at 973 K under 38 MPa in a vacuum.

### Measurements

The three-point flexural strength was measured at room temperature with a 30-mm span using specimens with dimensions of 3 × 4 × 40 mm at a crosshead speed of 0.5 mm/min. The fracture toughness was evaluated at room temperature according to the SEVNB (Single Edge V-notched Beam) method^15^ using V-notched specimens with a radius of curvature of 10–30 μm at the notch tip. These mechanical tests were conducted with the load direction parallel to the SPS-pressing direction.

HRTEM observations of the interface between disk-like Ni-Cr alloy particles and soda-lime glass matrix were performed using a Japan Electron JEM-2010F at UBE Scientific Analysis Laboratory Inc. The microstructure and fracture surface of the composites were observed by optical microscopy and scanning electron microscopy (Hitachi S-3200N).

The aspect ratio and spacing of disk-like particles in the cross-sectional microstructure perpendicular to the SPS-pressing direction were measured using analySIS FIVE (Olympus Corporation, Tokyo, Japan). The aspect ratio is defined as the ratio of the length of the major axis to the length of the minor axis in the circumscribed rectangles in each particle.

The bulk resistivity was measured by the van der Pauw method (Resitest 8300, Toyo Corporation, Tokyo, Japan) from room temperature to 600 °C under vacuum. The temperature coefficient of resistivity, α, was estimated from the following relationship:

$${\rm{\rho }}({\rm{T}})={{\rm{\rho }}}_{{\rm{0}}}\,[1+{\rm{\alpha }}\,({\rm{T}}-{{\rm{T}}}_{{\rm{0}}})]{\rm{.}}$$
